# Effect of ZnO on the pozzolanic activity and physico-mechanical properties of modified metakaolin cement mortar composites

**DOI:** 10.1038/s41598-025-34086-0

**Published:** 2026-01-16

**Authors:** M. A. Tantawy, M. A. Abdelzaher

**Affiliations:** 1 Chemistry department, Faculty of Science, Minia University, Minia, Egypt; 2 Environmental Science and Industrial Development Department, Faculty of Postgraduate Studies for Advanced Sciences, Beni-Suef University, Beni-Suef, 62511 Egypt

**Keywords:** ZnO-modified metakaolin (MMK), Surface modification, Pozzolanic activity, Strength activity index, Mortar, Chemistry, Engineering, Materials science

## Abstract

Metakaolin (MK) is commonly added to enhance the mechanical and durability properties of concrete. The pozzolanic activity of MK can be improved by calcining approximately ≈ 1 wt% ZnO with kaolin. However, the role of ZnO in enhancing the pozzolanic activity of MK remains unclear. The aim of this work is to investigate the surface modification of MK caused by ZnO that could enhance the pozzolanic activity of MK. ZnO-modified metakaolin (MMK) was prepared by calcination of kaolin powder (90 μm) with zinc carbonate basic equivalent to 1% by weight of ZnO, at 850 °C, and was analyzed by FTIR, XRD, and SEM techniques. The strength activity index according to ASTM C618, and the physico-chemical properties of blended cement mortars were measured at 28 days. Cement mortar samples were analyzed by FTIR, XRD, and SEM techniques. The XRD and FTIR results of MMK did not detect products of the interaction of ZnO and MK due to the detection limits. The SEM results illustrate the formation of uniform, non-aggregated (MMK) particles. The physico-chemical properties, strength activity index, FTIR, and XRD results of MK blended cement mortars indicated the higher pozzolanic activity of MMK. Whereas the SEM imaging shows the dispersion of cement particles coated with intense honeycomb-like C-S-H without being agglomerated in the case of MMK blended cement mortar. It was concluded that ZnO improves the pozzolanic activity by modifying the surface properties of MK during the calcination process as well as during the hydration process. The proposed mechanisms of surface modification of MK by ZnO were discussed. The addressed mechanism for visualizing the surface chemistry and microstructure of MMK paves the way for future studies on improving the pozzolanic activity of MK and the sustainability of cementitious-pozzolanic compositions.

## Introduction

Metakaolin that is obtained from the calcination of kaolin at a temperature of 650–850 °C is commonly added to enhance the mechanical and durability properties of concrete because of its pozzolanic activity^1^ and micro-filler characteristics^2^. Thermal treatment removes structural hydroxyl groups and collapses the ordered 1:1 Al–Si layers of kaolinite, producing a predominantly amorphous aluminosilicate (metakaolinite)^3^. This dehydroxylation alters aluminium coordination—octahedral OH environments are lost and Al adopts distorted four-, five- or mixed coordination—creating abundant reactive, non-crystalline Si and Al species^4^. Although the bulk is disordered, the surface of MK preserves (or rapidly regenerates in water) Si–OH and Al–OH groups that serve as the primary sites for adsorption, hydrogen bonding, and acid–base reactions^4^. Those surface hydroxyls make MK amphoteric: surfaces become positively charged at low pH and negatively charged at high pH, which governs zeta potential, ion uptake (e.g., Ca²⁺, Na⁺, Cl⁻), and dispersion in pore solution^5^. In the very alkaline environment of cement pore water, MK surfaces are generally negatively charged, promoting cation adsorption (notably Ca²⁺) and interactions with dissolved silicates and aluminates—behaviors that affect rheology (workability) and early hydration kinetics^4^. Finally, because finely ground MK has a high specific surface area, it increases water demand and strongly adsorbs organic admixtures, superplasticizers, and ions unless admixtures are used to compensate^6^.

The addition of MK enhances the early age compressive strength^7^, accelerates cement hydration^8^, improves the binding properties of blended cements^9^, reduces the pore size and porosity of cement pastes^2^, increases water absorption of concrete^10^, enhances the fire resistance of concrete^11^, improves the tensile and bending strength of concrete^12^, controls the deleterious expansion due to alkali-silica reaction in concrete^13^, reduces the chloride penetrability of concrete^11^, improves the sulfate attack resistance of concrete^14^, as well as reduces the creep and early age autogenous shrinkage of concrete^15^.

The pozzolanic activity of supplementary cementitious materials (SCMs) could be improved by mechanical grinding^16^, calcination^17^, acid treatment^18^, and addition of chemical activators^19^. Grinding technology is inefficient since about 95% of the energy input in the grinding process is lost to waste heat^20^. Calcination may activate SCMs such as clay if it produces an active amorphous calcined product. In contrast, calcination may deactivate SCMs if it decreases the surface area and/or increase the crystalline fraction of the calcined product^21^. Acid treatment increases the reactivity of SCMs due to the formation of gel film on their surface^18^ but it is limited for low-calcium SCMs^22^. Chemical activation is considered as the most effective and feasible method for the activation of SCMs because the addition of chemical activators even, could even maximize the material costs, but the targeted cost per unit strength development was minimized^23^.

There are some research articles on the preparation of active MK by calcination of kaolin with 0.1–1 wt% ZnO. Sánchez et al. 2020^24^ found that calcination of kaolin at 600 °C for 2 h with doping with 1% zinc oxide represented ideal conditions for thermal/chemical activation of kaolin. This improved the pozzolanic activity of the MK produced and supported the formation of more stable hydrolyzed products, which enhanced the durability and mechanical properties of the cement products to which it was added. It was claimed that ZnO does not change the percentage of dehydroxylation of kaolin or the structure of MK. Recent studies prepared activated calcined kaolin, montmorillonite, and illite shale by calcination with 0.1–1 wt% ZnO. It was confirmed that the combination of ZnO and MK eliminates the retardation effect caused by ZnO and even accelerates the hydration of cement^25,26^. However, the role of ZnO in the promotion of the pozzolanic activity of MK is not yet clearly explained, possibly because the elucidation of the chemical and microstructural changes resulting from the combination of ZnO and MK has not been detected by XRD, FTIR, and SEM techniques^25–27^ particularly because of the limited amount of ZnO. Instead, researchers tried to explain the effect of ZnO on cement hydration and applied theories that explain how ZnO retards and/or re-initiates the process of cement hydration despite the low amount of ZnO added.

To understand the whole picture, we need to address these theories. It has been agreed that the addition of ZnO retards the hydration of cement by elongating the induction period, then re-initiates the hydration again^28^. There are two different views on the interpretation of the mechanism by which ZnO retards the hydration of cement. The first mechanism assumes that ZnO dissolves and precipitates in cement pore solution forming, amorphous Zn(OH)_2_ that coats the anhydrous cement grains and retard its hydration. Then, Ca^2+^ ions react with the amorphous Zn(OH)_2_ coating layer forming crystalline calcium zinc hydroxide (CaZn_2_(OH)_6_.2H_2_O); as a result, the hydration of cement is resumed^29,30^ which may improve the mechanical properties of cement paste^31^. Conversely, according to^32^, detection of calcium zinc hydroxide phase in paste samples containing ZnO at the early age of hydration without any evidence for the presence of a Zn(OH)_2_, proves that this mechanism is empirically uncertain.

The second mechanism assumes that cement hydration has been retarded by the dissolution of ZnO in the pore solution, which is accompanied by removed of Zn^2+^ ions by precipitation of calcium zinc hydrate and adsorption of Zn^2+^ ions on C-S-H nuclei a, state called C-S-H poisoning. This retardation effect ends when the rate of Zn^2+^ removal exceeds the rate of dissolution^33^. This means that ZnO is considered as a delayed accelerator^34^. It was observed that the addition of the SCMs, such as MK, suppresses the retardation effect of ZnO^25,33^ because the SCMs could adsorb and lower the concentration of Zn^2+^ ions, which causes C-S-H poisoning. In addition to that, SCMs act as nucleation sites for C-S-H growth that suppresses retardation caused by Zn^2+^ ions^35^.

Accordingly, ZnO by acting as a hydration retarder, could be added to concrete in special applications^29,36^. In contrast, ZnO is commonly used to improve the concrete durability properties and to develop what is called a functionalized concrete. Although the addition of ZnO nanoparticles slightly increases the water requirement, it elongates the setting time and retards the hydration of the cement paste. In contrast, ZnO nanoparticles improve the compressive strength of cement paste due to the filler effect^30^. The addition of ZnO nanoparticles was also found to improve the workability of concrete by competing with cement particles in the adsorption of the superplasticizer^37^. ZnO was found to be effective in reducing the corrosion rate of the reinforcing steel bars in concrete^36^. The addition of ZnO nanoparticles was found to improve the photocatalytic properties of concrete to be used in self-cleaning applications^38,39^. The addition of 15 mass % ZnO nanoparticles to cement possesses effective antibacterial and antifungal activity of the cement composite, which represents a giant leap towards the development of functionalized cement-based materials^40^.

The aim of this work is to investigate the role of ZnO in the promotion of the pozzolanic activity of MK and proposing a mechanism of surface modification by ZnO on the activity of MK. MMK was prepared by calcination of kaolin powder (90 μm) with zinc carbonate basic equivalent to 1% by weight of ZnO, at 850 °C, and was analyzed by FTIR, XRD, and SEM techniques. The strength activity index, compressive strength, combined water content, bulk density, and total porosity of blended cement mortars containing 75 wt% of sand, 20 wt% Ordinary Portland cement (OPC), and 5 wt% MK with a w/c ratio of 0.5, were measured up to 28 days. The mortar was used instead of neat cement paste in this study because it more accurately represents practical cementitious systems, where aggregates are always present, and therefore provides more application-friendly results.

## Materials and experimental

**Fig. 1:**
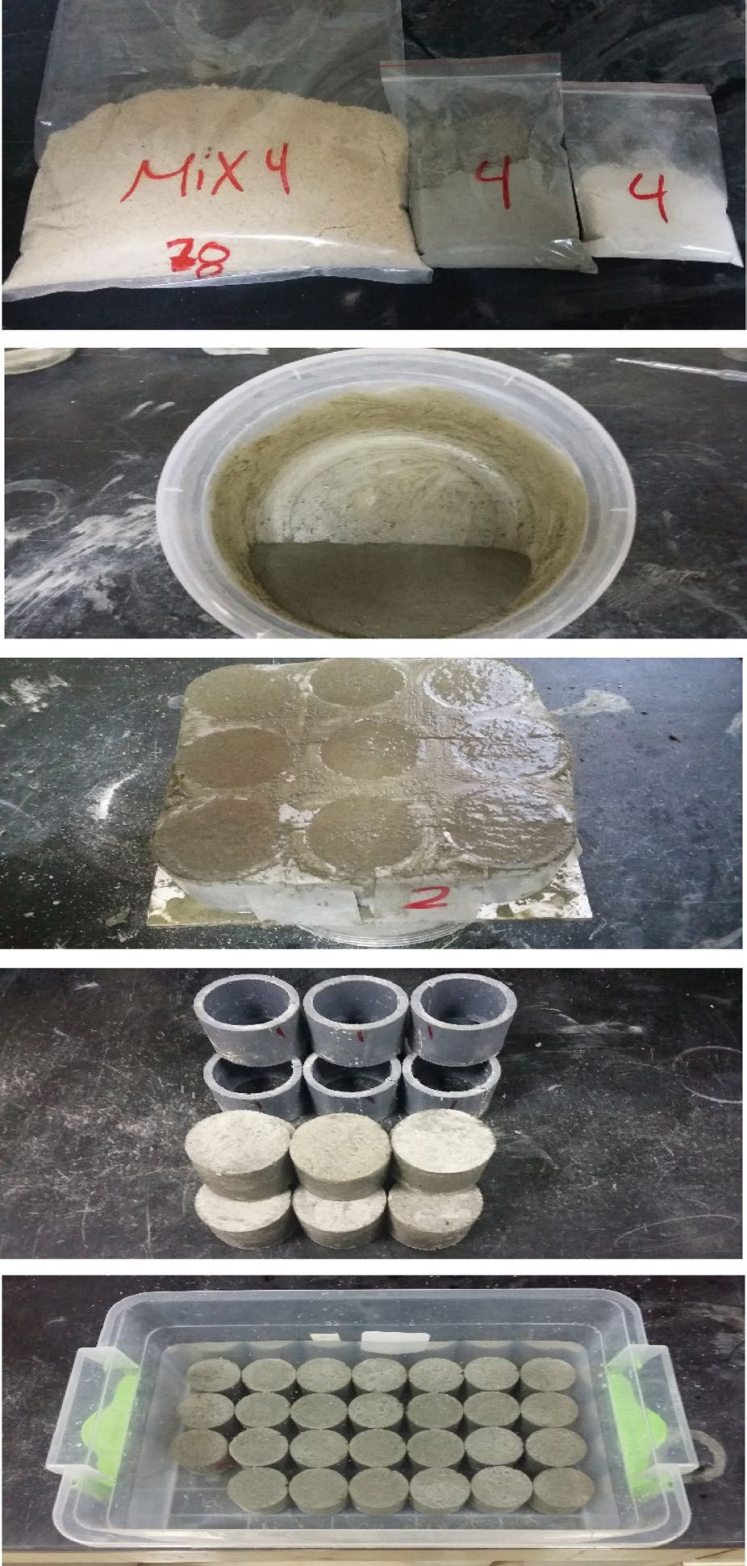
Cement mortar preparation, casting and curing.

A commercial Egyptian kaolin sample was crushed, ground to a very fine powder using a lab scale-ball mill, and passed from a sieve with an opening of 90 µm. Kaolin powder was well mixed with zinc carbonate basic [ZnCO_3_]_2_.[Zn(OH)_2_]_3_ (BDH Chemicals Ltd, Poole, England) equivalent to 1 wt% ZnO and calcined in a muffled furnace at 850 °C for 2 hours. MMK was immediately removed from the furnace, cooled down, and ground to pass a 90 µm sieve. The samples of kaolin, MK, and MMK are symbolized as K, MK, and MMK, respectively. The pozzolanic activity of MK and MMK was evaluated by the strength activity index test, ASTM method^41^. Dry blended cement mix contains 75 wt% sand, 20 wt% OPC [CEM 1, 42.5], and 5 wt% MK or MMK, and a control cement mix contains 75% sand and 25% OPC. Cement mortars were prepared by mixing the dry mix with water (w/c ratio of 0.5) in a mixer for 5 minutes and were cast in 1.5’’ diameter cylinders. Fresh mortar cylinders were covered with a plastic sheet and cured in a humid chamber, de-molded after 24 hours, and immersed in a water tank at 23 °C (Fig. 1). At the age of 3, 7, and 28 days, three identical mortar specimens were tested for compressive strength, bulk density, total porosity, as well as, the free, combined, and total water content measurement. Compressive strength of cement mortars was tested by a compressive strength machine according to ASTM method^42^. Load was applied at 8–16 kgf/cm²·s for 1.5’’ diameter cylinders, and the rate was held constant during the first half of the anticipated maximum load and unchanged during the remainder of the test. The free water content of cement mortars was determined using a microwave oven^43^. The compressive strength test residue was crushed, and approximately 5 g of weight was taken into a watch glass, placed in a microwave oven, and heated at 100% radiation for 2 min.

The sample was then cooled in a desiccator before being reweighed. The free water content was measured from the weight difference before and after heating in the microwave oven. The combined water content of cement mortars was determined by igniting the microwave dried specimens in a muffled furnace at 1000 °C for 1 h^44^. Bulk density of cement mortars was evaluated according to Archimedes’ principle^45^. Measurement of the total porosity of cement mortars was performed as described elswhere^46^.

K, MK, and MMK, as well as cement mortar samples, were analyzed by FTIR, XRD, and SEM techniques. The chemical composition of MK was analyzed by a Philips PW1606 XRF spectrometer. The content of minerals was analyzed by Philips X-ray diffractometer PW 1370 with Ni-filtered CuK_α_ radiation (1.5406 Å). The semi- quantitative analysis of phases appearing in XRD was calculated using the Bruker AXS configuration program. FTIR analysis was performed using the spectrometer Perkin Elmer FTIR System Spectrum X at a range of 400–4000 cm^- 1^. TGA and DTA were measured by Netzsch STA 409 C/CD analyzer at 2 °C/min heating rate from room temperature up to 1000 °C, under air atmosphere at 50 ml/min flow rate. SEM was analyzed by Jeol-Dsm 5400 LG apparatus with an elemental chemical composition detecting probe, EDX.

## Results

**Fig. 2:**
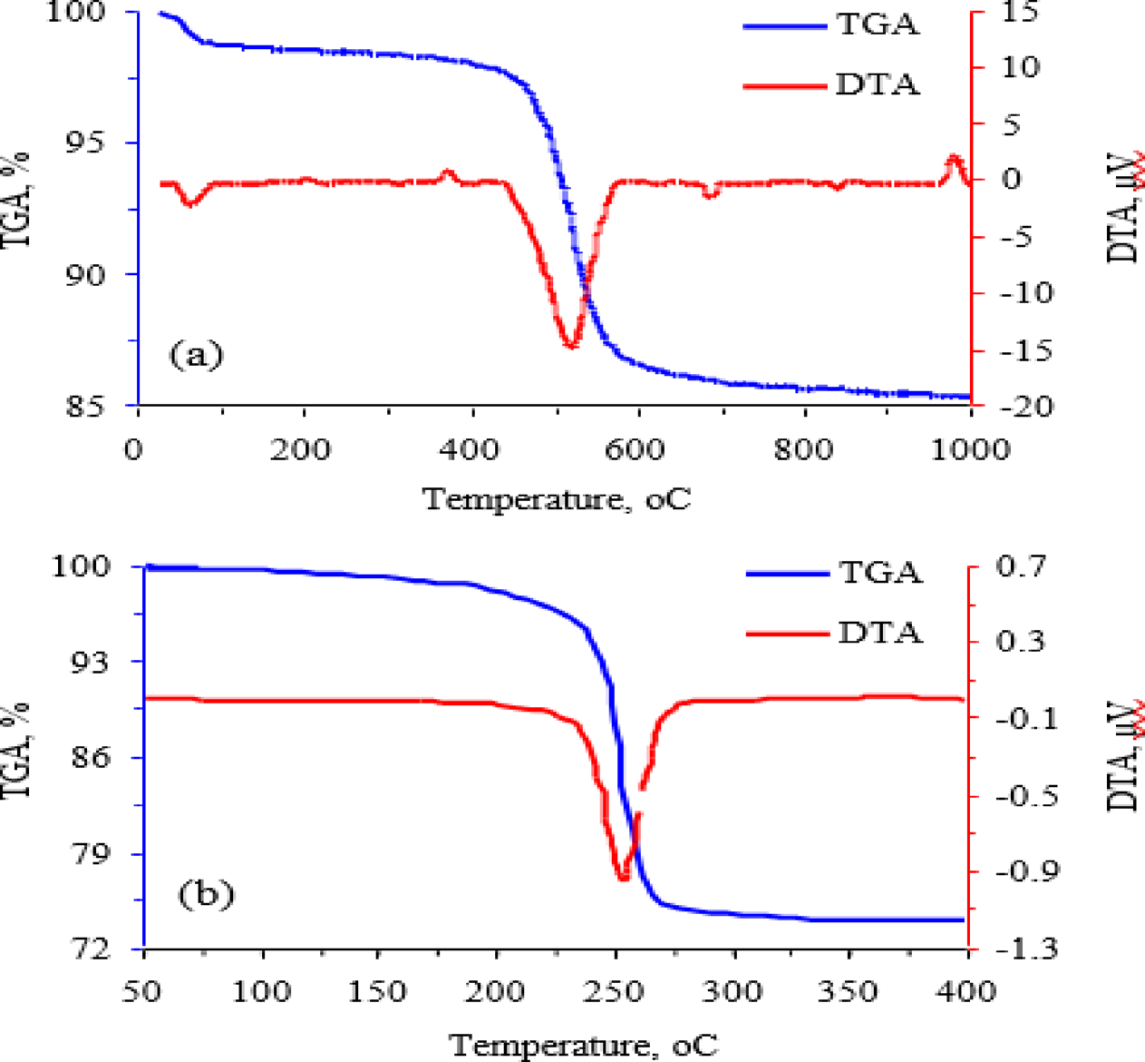
TGA and DTA of (**a**) kaolin and (**b**) zinc carbonate basic.

TGA analysis of kaolin (Fig. 2) shows that a small percentage weight loss occurs before 200 °C due to loss of moisture absorbed by kaolin particles, and 11 wt% weight loss occurs at 350–700 °C, accompanied by the endothermic peak at 520 °C, due to the removal of the structural water of the kaolinite mineral and formation of metakaolinite^47^. DTA analysis of kaolin shows the sharp exothermic peak at 981 °С due to the transformation of metakaolinite to Al-Si spinel or a mixture of γ-alumina, amorphous silica, and mullite^48^. TGA analysis of zinc carbonate basic (Fig. 2) illustrates that 25.9 wt% weight loss occurs at 175–325 °C, accompanied by the endothermic peak at 254 °C, due to the decomposition of zinc basic carbonate into ZnO^49^.

The content of the oxides of MK inferred by XRF analysis (Table 1) shows that MK contains 42.1 wt% Al_2_O_3_, 51.3 wt% SiO_2_, 1.2 wt% TiO_2_, 1.8 wt% Fe_2_O_3_, and trace amounts of CaO, MgO, Na_2_O, and K_2_O oxides.

**Table 1:** XRF analysis of OPC and MK.

Oxide, Wt %	SiO2	Al2O3	CaO	Fe2O3	MgO	SO3	Na2O	K2O	TiO2	Cl^-^	LOI	Total
**OPC**	20.88	6.08	63.11	3.18	1.51	1.60	0.22	0.24	0.20	0.12	2.35	99.49
MK	51.33	42.13	0.57	1.82	0.30	0.23	0.22	0.50	1.27	0.07	1.42	99.86

**Fig. 3:**
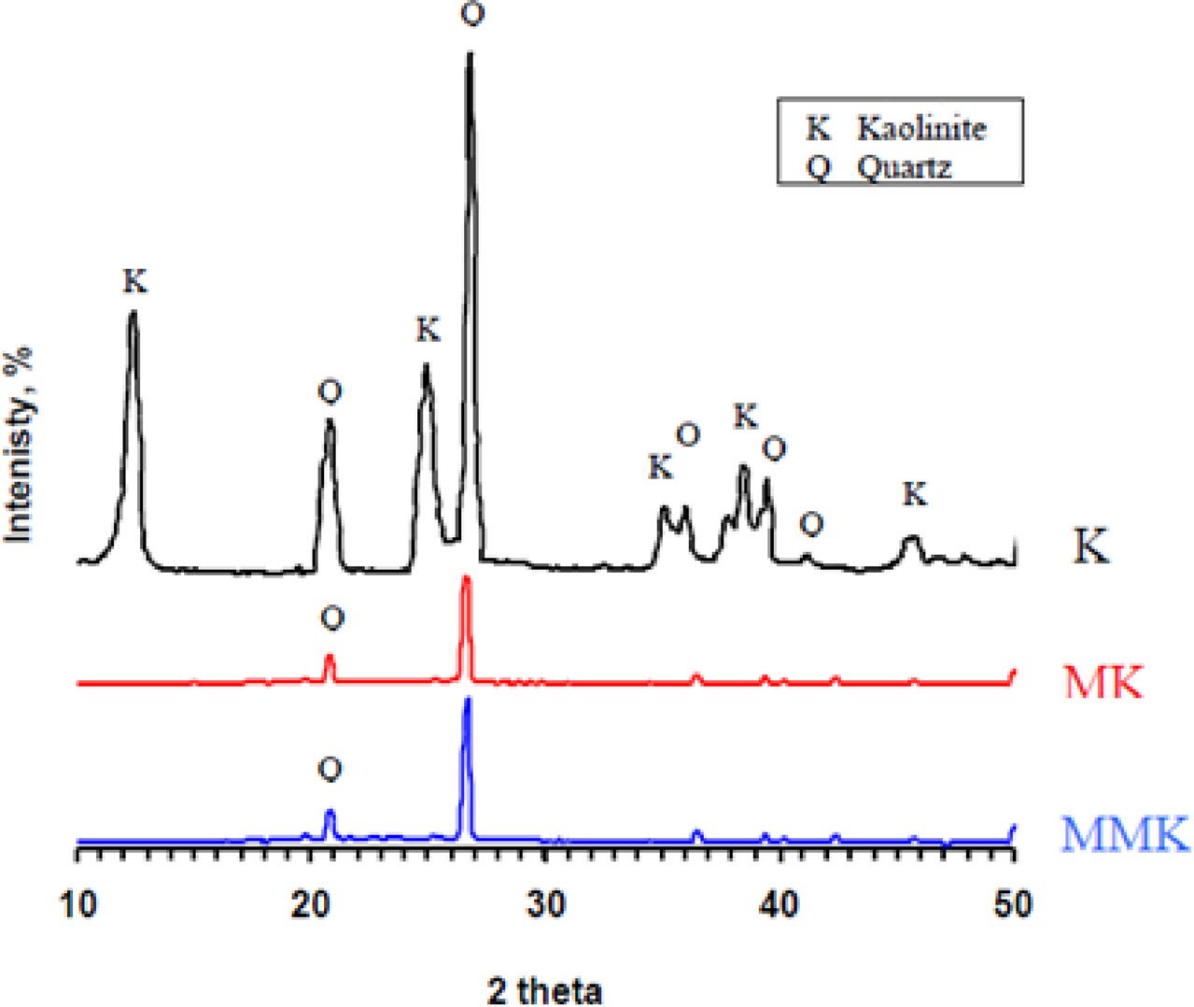
XRD patterns of K, MK, and MMK.

The XRD results of kaolin, MK, and MMK (Fig. 3) prove that kaolin contains 67.0 wt% kaolinite (Al_2_O_3_.2SiO_2_.2H_2_O), 29.5 wt% quartz (SiO_2_), and a trace amount of anatase (TiO_2_). MK contains quartz and amorphous phases of aluminosilicates that arise from the dehydroxylation of the kaolin. The structure of MMK is the same as the MK. The formation of ZnO could not be evidenced by XRD analysis. The XRD result was not surprising, given that the conventional XRD apparatus cannot detect minerals that make up less than about 5% of the sample size. As raw kaolin contains 1 wt% ZnO, the percent of ZnO does not exceed this limit. Accordingly, the characteristic diffraction lines of ZnO will be hardly detected in the diffraction pattern^50^.

**Fig. 4:**
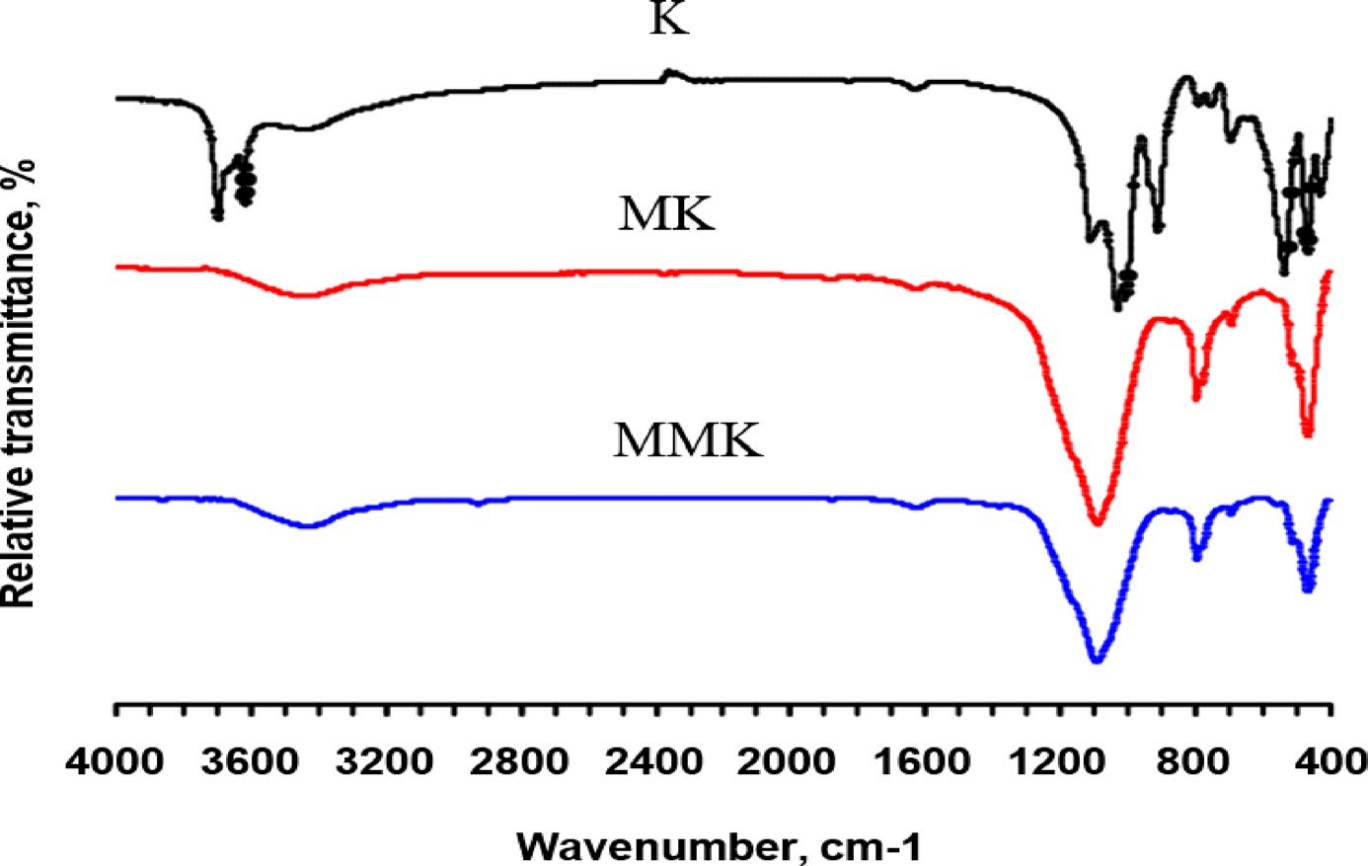
FTIR spectra of K, MK, and MMK.

The results of the FTIR analysis of kaolin, MK, and MMK (Fig. 4; Table 2) are consistent with XRD results, confirming that kaolin contains kaolinite and quartz. Vibration bands appearing at 532, 908, 1025, 1105, 3618, and 3700 cm^- 1^ are attributed to the functional groups of the kaolinite structure^51,52^. Bands appearing at 465 and 683 cm^−1^ are attributed to the functional groups of the quartz structure^53^. While bands appearing at 3407 and 1610 cm^−1^ are attributed to the (OH^-^) group of adsorbed water molecules^54^. The result of the FTIR spectrum confirms that MK contains quartz and metakaolinite. the band appearing at 1090 cm^−1^ is attributed to the amorphous SiO_2_ arising from loss of the crystalline hydroxyl groups and disorder of the sheet structure of kaolinite as a result of calcination^55^. Also, FTIR analysis was not able to confirm the nature of the new compounds that could result from the reaction of ZnO and MK because most spectrochemical techniques, such as FTIR, face significant practical detection limits for traces present in the sample, especially for materials that poorly absorb infrared radiation or when spectral bands of the trace material coincide with those of major components^56^.

**Table 2:** Description of FTIR spectra of K, MK, and MMK.

No.	Spectrum	Band cm-1	Band description	Structure	Refs.
1	Kaolin (K)	532	vibration of Al-O-Si octahedral	kaolinite	^51^
2		908	bending vibration of Al-O-H		
3		1025	alternating stretching vibration of Si-O-Si and Al-O-Al		
4		1105	asymmetric stretching vibration of Si-O-Si -		
5		3618	vibration of internal OH groups -		^57^
6		3700	elongation vibration of edge OH groups		
7		465	bending vibration of O-Si-O	quartz	^53^
8		683	symmetric stretching vibration of Si-O-Si		
9		1610	bending vibration of HO^-^	adsorbed water	^54^
10		3407	stretching vibration of HO^-^		
12	Metakaolin (MK)	459	bending vibration of O-Si-O	quartz	^53^
13		686	symmetric stretching vibration of Si-O-Si		
14		1090	stretchingvibrationof Si-O (amorphous SiO_2_)	metakaolinite	^55^
15		1610	bending vibration of HO^-^	adsorbed water	^54^
16		3407	stretching vibration of HO^-^		
17	ZnO- modified metakaolin (MMK)	459	bending vibration of O-Si-O	quartz	^53^
18		686	symmetric stretching vibration of Si-O-Si		
19		1090	stretchingvibrationof Si-O (amorphous SiO_2_) -	metakaolinite	^55^
20		1610	bending vibration of HO	adsorbed water	^54^
21		3407	stretching vibration of HO^-^		

**Fig. 5:**
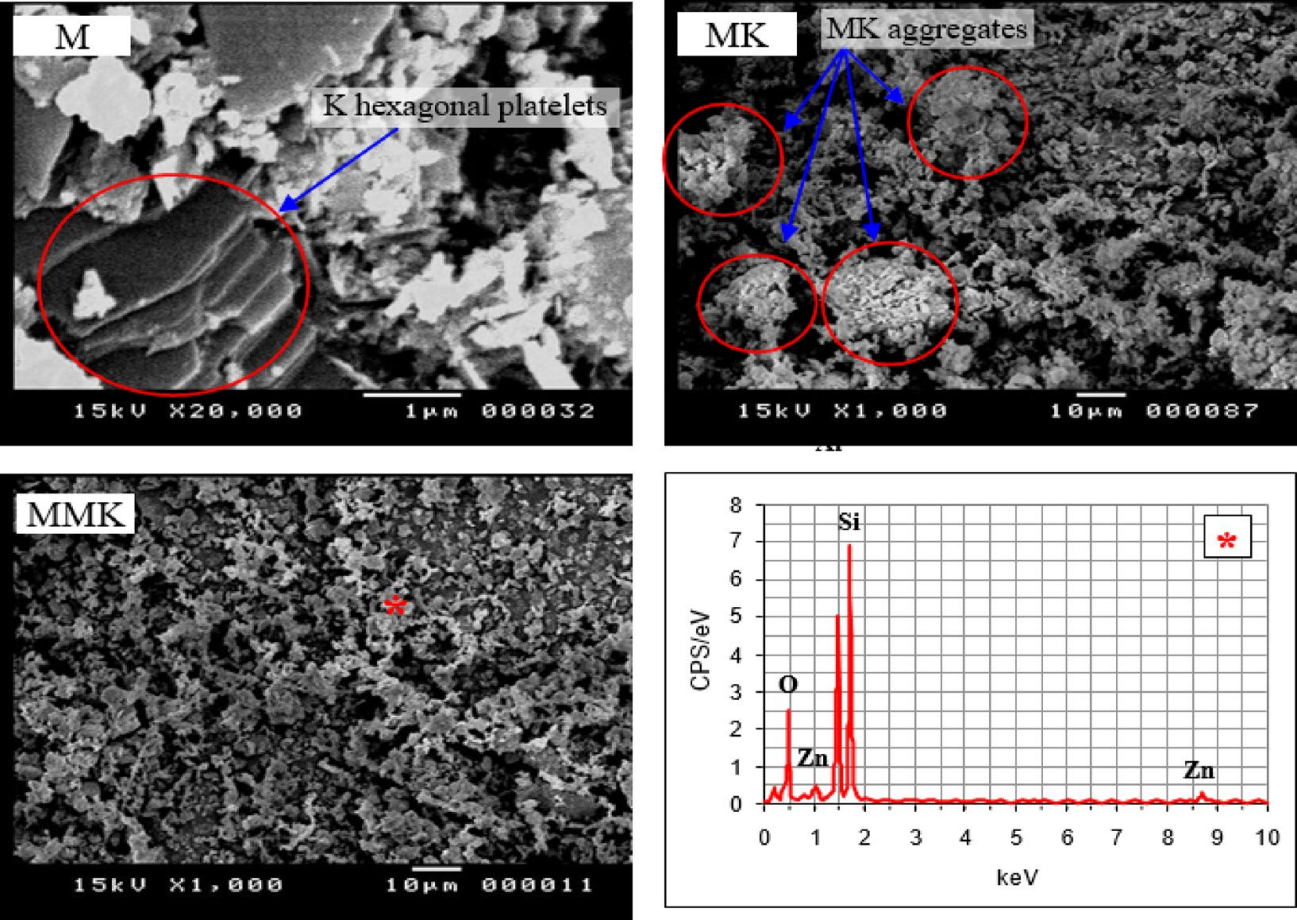
SEM photographs of K, MK, and MMK as well as EDX of MMK.

The SEM micrographs of kaolin, MK, and MMK (Fig. 5) were provided with EDX analysis of MMK. The SEM micrograph of kaolin was performed at high magnification to show flat hexagonal platelets of kaolinite stacked together. The SEM micrographs illustrate the change in the particle size and specific surface area of the produced MK and MMK due to the effect of calcination and ZnO addition, respectively. At the beginning of calcination, the kaolinite particles contract due to the loss of water, giving MK with a smaller particle size and higher specific surface area. Then the kaolinite particles could be aggregated due to sintering of MK particles, giving MK with a larger particle size and lower specific surface area^21,58^. The SEM micrograph ZnO-modified MK shows the existence of very small white particles adhering to MK grains. EDX analysis of the MMK sample demonstrates that the sample contains ZnO due to the appearance of two Zn peaks. During the analysis, the selection of the target area to measure the proportion of chemical elements was repeated so as to obtain the measurement that shows the presence of an appreciable percent of ZnO. The SEM micrograph of MK also shows the existence of aggregated lumps due to sintering of the MK. In contrast, the SEM micrograph of MMK shows the existence of uniformly non-aggregated MK particles. This proves that ZnO particles retard the sintering and aggregation of MK particles.

**Fig. 6:**
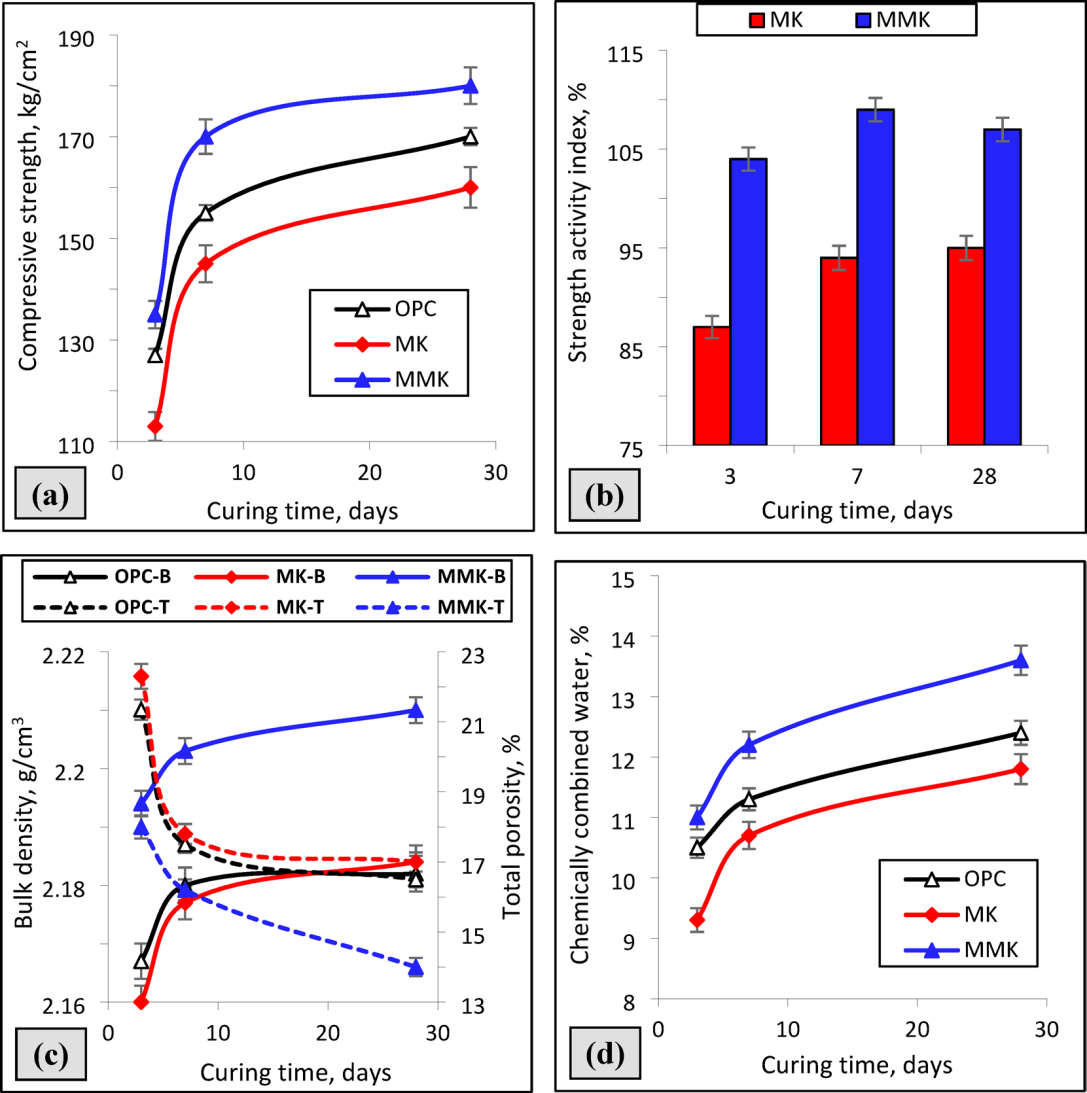
Compressive strength (**a**), strength activity index (**b**), combined water content (**c**), bulk density and total porosity (**d**) of OPC as well as MK and MMK blended cement mortars cured for 28 days.

The compressive strength, strength activity index, combined water content, bulk density, and total porosity of OPC, as well as MK and MMK blended cement mortars (Fig. 6) were tested for 28 days of hydration. Results of the compressive strength, combined water, and bulk density increase, whereas the total porosity decreases with curing time due to the progress of hydration reactions leading to the formation of additional C-S-H gel, which precipitates and fills some of the open pores inside hardened cement mortars^59^. Results of the compressive strength, combined water content, bulk density, and total porosity developed rapidly in the first few days of hydration of OPC and other blended cement mortars. The compressive strength, combined water content, and bulk density of MMK cement mortar are higher than those of OPC mortar. In contrast, the compressive strength, combined water content, and bulk density of MK blended cement mortar are lower than those of OPC mortar. This proves that MK possesses filler and dilution effects on the hydration of cement mortar as it replaces a part of OPC^2,60^, whereas MMK possesses higher pozzolanic activity. The reactive and amorphous MMK particles react with the portlandite that is liberated from the hydration of cement, forming more C-S-H gel, which precipitates and fills some of the open pores inside hardened cement mortars^59^. The ASTM C618 specification assumes that a positive strength activity index for a specific pozzolana is achieved when the strength activity index result is greater than 0.75 after 7 and 28 days for the cement mortar containing 20% of the pozzolana^61^. The results showed that the strength activity index of MK and MMK blended cement mortars are 0.95 and 1.07 after 28 days of hydration, respectively. According to this, both MK and MMK have a positive index of pozzolanic activity, but the latter is better because of the role of ZnO in modifying the surface and pozzolanic properties of MK.

**Fig. 7:**
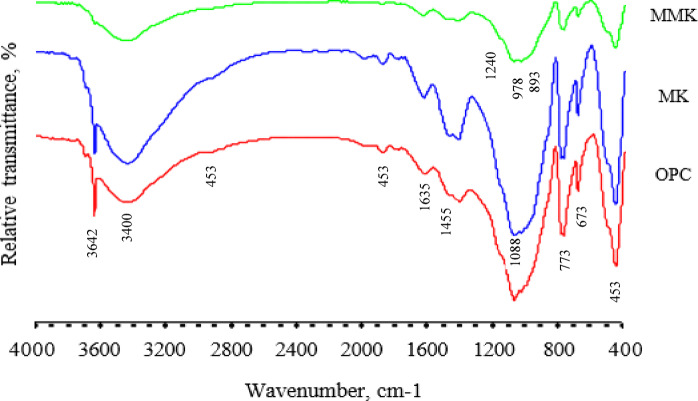
FTIR spectra of OPC as well as MK and MMK blended cement mortars.

The FTIR analysis of OPC, MK, and MMK blended cement mortars (Fig. 7) explains the chemical reactions that occur during hydration of OPC, as well as MK and MMK blended cement mortars. Bands appearing at 453, 773, and 1088 cm^−1^ are attributed to the O-Si-O bending as well as Si-O-Si symmetric and asymmetric stretching vibrations in quartz sand^62^. The broad band of Si-O-Si asymmetric stretching vibration of quartz appearing at 850–1250 cm^–1^ prevented the appearance of the characteristic bands of calcium silicate hydrate C-S-H at 978 cm^−1^, which represents the asymmetric stretching vibration of SiO_4_^4–63^, and prevented the appearance of the ettringite band at 1124 cm^−1^. An unhydrated alite and belite phase usually has a shoulder appearing at 498 cm^−1^, which is attributed to the O-Si-O symmetric stretching vibration, and a band at 877 cm^- 1^, which is attributed to the O-Si-O asymmetric stretching vibration. The latter band overlaps with the broad band of Si-O-Si asymmetric stretching vibrations in quartz. The intensity of this band markedly decreased in the case of MMK blended cement mortar. This proves the difference in pozzolanic activity between MK and MMK and shows that the later has higher pozzolanic activity. Broad band appearing at 1050–1150 cm^−1^ is attributed to the symmetric and asymmetric stretching vibration of SO_4_^2-^ in gypsum phase^64^. The intensity of this band markedly decreased and was shifted to a lower value in the case of MMK blended cement mortar, due to the formation of the ettringite phase. MK reacts with gypsum and portlandite, forming ettringite because MK is more soluble than tricalcium aluminate C_3_A^65^. This indicates that MMK is more likely to be involved in this reaction (i.e., more soluble) than MK. The band appearing at 1455 cm^−1^ is attributed to the asymmetric stretching vibrations of CO_3_^2-^ in calcite phase^66^. Whereas the band of the out-of-plane bending vibration of CO ^2-^ which appears at 893 cm^- 1^ overlap with the broad band of Si-O-Si asymmetric stretching vibrations in quartz.

The intensity of this band can be taken as an indicator of the extent of carbonation of cement mortar by water-dissolved CO_2_. The intensity of these bands increases in the case of MK blended cement mortar, then decreases in the case of MMK blended cement mortar. This result is fully compatible with total porosity results that showed that MMK blended cement mortar is less porous than MK blended cement mortar. The band appearing at 1635 cm^−1^ is attributed to the stretching vibration of water molecules associated with calcium silicate hydrate C-S-H^67^. While the band appearing at 3400 cm^- 1^ is attributed to the stretching vibration of OH^-^ in free water molecules^62^. The band appearing at 3642 cm^- 1^ is attributed to the stretching vibration of OH^-^ in the portlandite phase. The intensity of this band markedly decreased in the case of MMK blended cement mortar. This indicates the high pozzolanic activity of MMK.

**Fig. 8:**
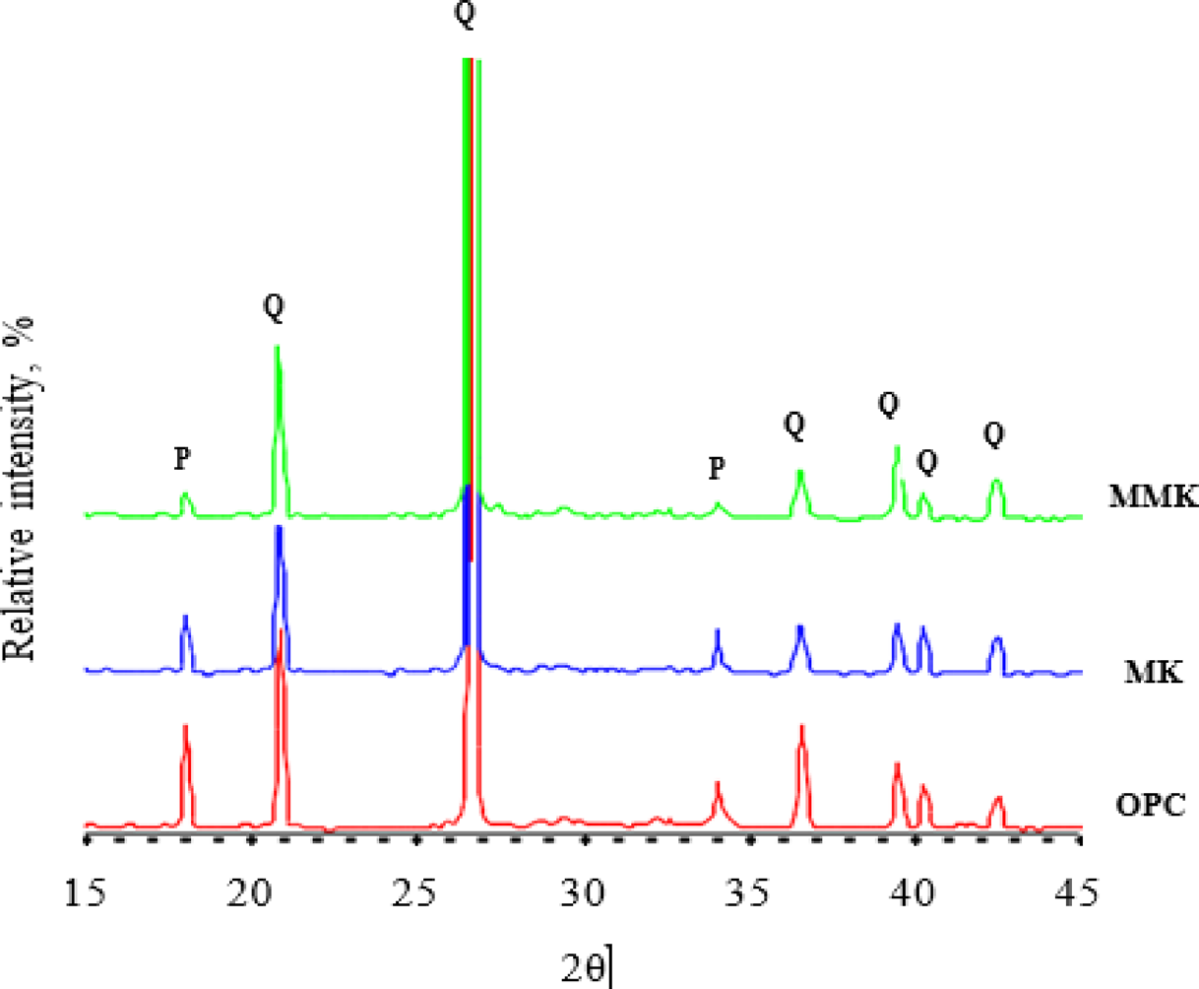
XRD patterns of OPC as well as MK and MMK blended cement mortars. P portlandite and Q quartz.

The XRD analysis of OPC, MK, and MMK blended cement mortars (Fig. 8) shows the relative percentages of crystalline phases involved in the hydration process of OPC, as well as MK and MMK blended cement mortars. It is clear that the major phase is portlandite besides quartz. The relative percent of quartz is similar in all cement mortars due to the fixed ratio of sand that was added in all mortar formulations. Whereas the relative percent of portlandite decreases in the case of MK blended cement mortar and decreases further in the case of MMK blended cement mortar. This confirms the order of strength activity index, that MMK has more activity than MK, as measured by the strength activity test. The modification of MK by ZnO improves the pozzolanic activity of MMK. Hence, MMK reacts with more portlandite, forming more and more of C-S-H.

SEM micrograph of MMK (Fig. 5) exhibits a more dispersed, less-aggregated morphology compared with unmodified MK, shifting toward smaller primary particle sizes. This is consistent with the hypothesis that ZnO inhibits grain growth during calcination. EDX elemental analysis (Fig. 5) shows clear Zn signals co-located with Si/Al on the surface of modified particles, supporting surface doping rather than separate coarse ZnO agglomerates. These morphology-chemistry-linked observations explain the higher pozzolanic activity measured by the strength activity index (Fig. 6b) and the greater portlandite consumption shown in FTIR/XRD results (Figs. 7 and 8). Smaller, well-dispersed ZnO-modified MK particles react more rapidly with Ca(OH)₂ to form C–S–H and aluminosilicate hydrates, thereby improving early and 28-day reactivity (Fig. 6a).

**Fig. 9:**
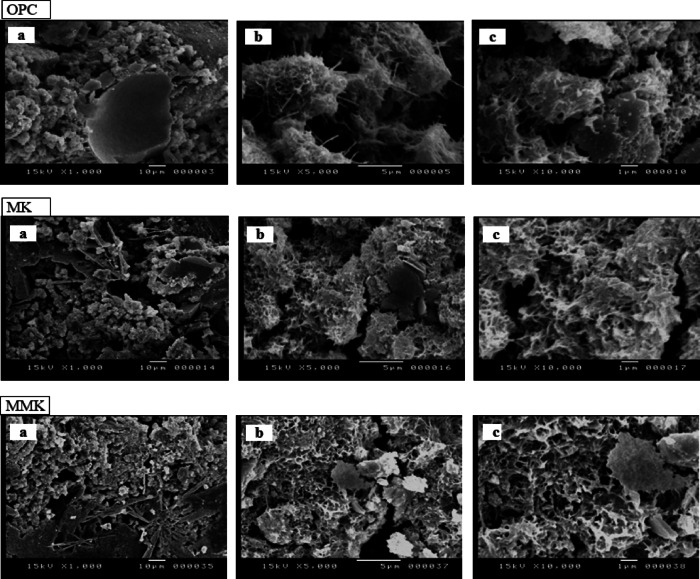
SEM photographs of OPC as well as MK and MMK blended cement mortars.

The SEM analysis of OPC, MK, and MMK blended cement mortars (Fig. 9) illustrates the changes that occur in the microstructure of cement mortars during hydration of OPC, as well as MK and MMK blended cement mortars. The SEM micrograph of OPC mortar shows the appearance of little fibrous C-S-H that coats the unhydrated cement grains. The SEM micrograph of MK blended cement mortar shows the formation of honeycomb- like C-S-H coating agglomerated cement particles. In contrast, the imaging of MMK shows well-dispersed cement particles coated with intense honeycomb-like C-S-H without being agglomerated. This proves that modification of MK by ZnO improves the dispersion and prevents agglomeration of MMK particles inside the cement matrix, as well as improves its pozzolanic activity.

## Discussion

TGA/DTA results (Fig. 2) prove that during the calcination step, zinc carbonate basic decomposes to ZnO (Eq. 2) in the temperature range 175–325 °C by removal of CO_2_ and water. Whereas kaolin decomposed to MK (Eq. 2) in the temperature range 350–700 °C via the removal of the structural water of the kaolinite mineral.

1 $$Z{n_5}{\left( {C{O_3}} \right)_2}{\left( {OH} \right)_{6(s)}} \to 5Zn{O_{(s)}} + 2C{O_{2(g)}} \uparrow + 3{H_2}{O_{(g)}} \uparrow$$

2 $$A{l_2}{O_3}^.2Si{O_2}^.2{H_2}{O_{\left( s \right)}} \to A{l_2}{O_3}^.2Si{O_{2(s)}} + {\text{ }}2{H_2}{O_{(g)}} \uparrow$$

The XRD and FTIR techniques (Figs. 3 and 4) did not detect the change in chemical and structural composition of MK and MMK i.e., it did not detect the existence of ZnO or any phases that could arise from the interaction of ZnO and MK because of the detection limits, in contrast, these techniques show that the structure of MK and MMK are similar. On the other hand, the SEM technique (Fig. 5) could help to detect the change in morphology and surface characteristics of MK and MMK. The SEM technique of MK proves the formation of aggregated MK particles, whereas, the MMK particles are non-aggregated. The physico-chemical properties (Fig. 6, compressive strength, combined water content, and bulk density) of MMK blended cement mortar are higher than OPC mortar, whereas those of MK blended cement mortar are lower than OPC mortar. The FTIR and XRD results (Figs. 7 and 8) of MK blended cement mortars indicated the higher pozzolanic activity of MMK because of the role of ZnO in modifying the surface as well as pozzolanic properties of MK.

FTIR results illustrated another difference in chemical activity between MK and MMK. The result proves that MMK could be more responsible than MK in reaction with gypsum and portlandite (Eq. 3) to form ettringite^65^:

3 $$Al2O3.2SiO2+3CaSO4.2H2O+3Ca(OH)2+27H2O{\rightarrow}Ca6Al2(SO4)3.(OH)12.26H2O+2SiO2.2H2O$$

The SEM technique (Fig. 9) could be helpful in comparing the microstructure, homogeneity, consistency, and dispersion characteristics of different blended cement mortars. SEM result of MK shows the formation of honeycomb-like C-S-H coating agglomerated cement particles, whereas that of MMK shows the dispersion of cement particles coated with intense honeycomb-like C-S-H without being agglomerated.

The observations obtained from the SEM results of MK and MMK, as well as their blended mortars, open the way for an attempt to explain the role of ZnO in preventing the aggregation of MMK particles during calcination, as well as improving the dispersion of MMK cement matrix during hydration of cement mortar. This way, in fact, depends on the assumption that the interaction of ZnO with MK particles during the calcination process induces modification of the surface of MK by ZnO particles. The mechanism of surface modification of MK by ZnO could be the key to understanding the role of ZnO in improving the pozzolanic activity of MK. The mechanism may be divided into two separate parts: (i) the mechanism of surface modification in the calcination step and (ii) the mechanism of surface modification in cement pore solution during the hydration process.

**Fig. 10:**
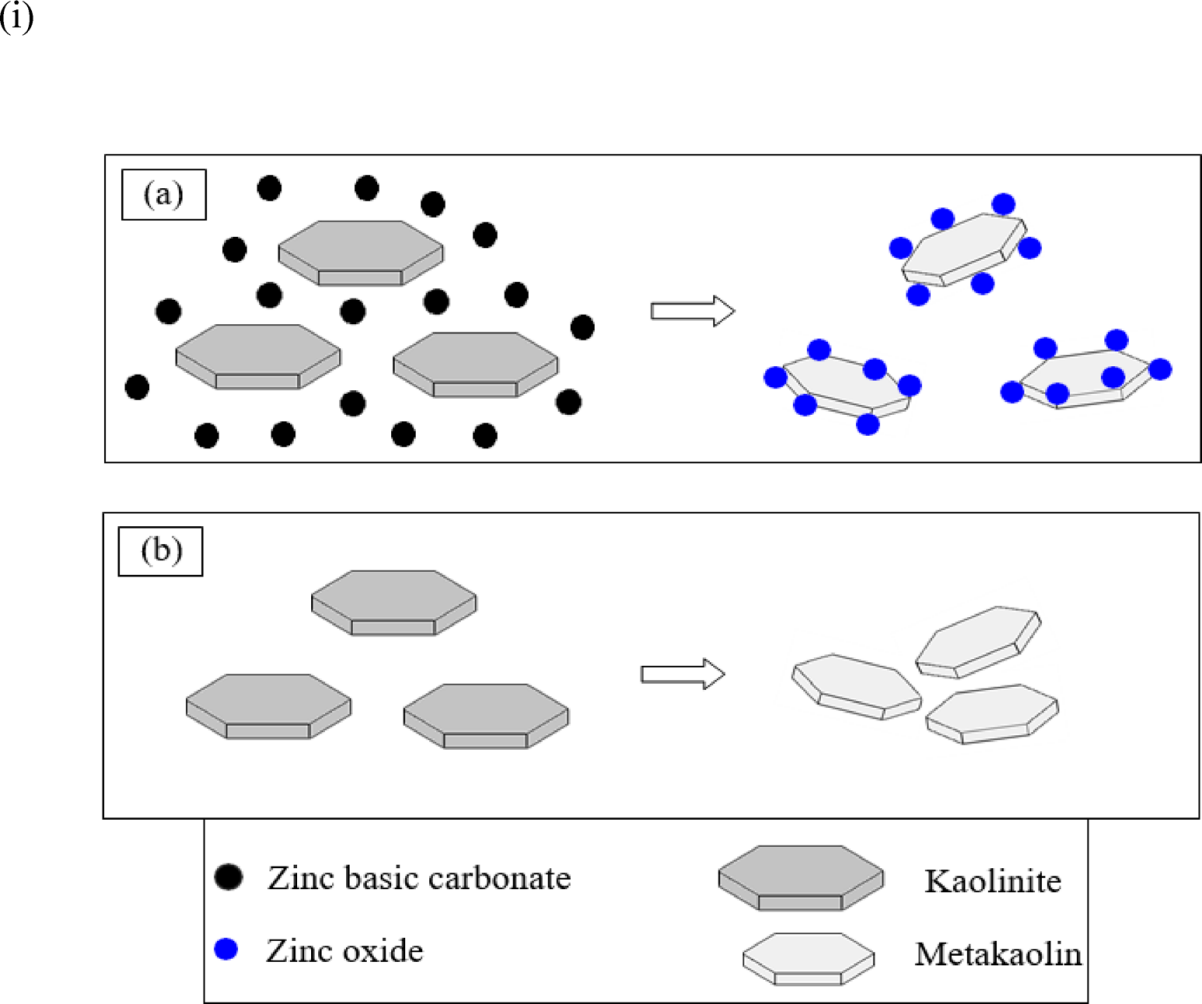
The proposed mechanism by which ZnO may modify the surface of MK in the calcination process (**a**) in presence and (**b**) in absence of zinc precursor.

**Fig. 11:**
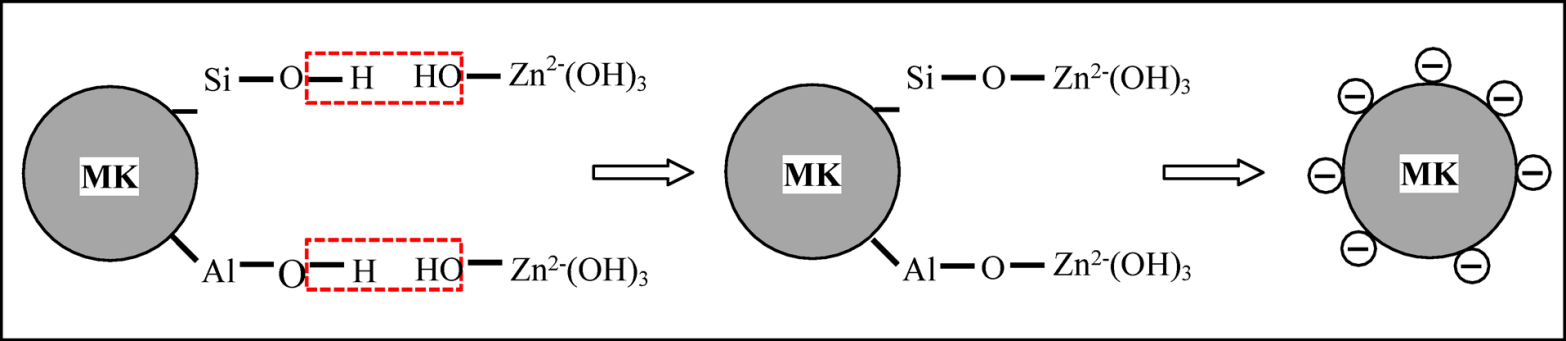
The proposed mechanism by which ZnO may modify the surface of MK inside the cement pore solution.

(i) The mechanism of surface modification of MK by ZnO in the calcination process (Fig. 10) assumes that doping with ZnO leads to the formation of MK having the high specific surface area and pozzolanic activity, as illustrated in the following steps:

1. Zinc carbonate basic decomposes during calcination with kaolin, forming ZnO in the temperature range 200–260°C^49^. The produced ZnO grows on, coats the surface of dehydroxylated kaolinite crystals, and bonds to the aluminosilicate groups of MK surface^67^, producing MK particles coated with a ZnO layer.
2. Depending on the calcination conditions of kaolin, the de-hydroxylated kaolinite particles may tend to contract due to the loss of water (giving MK with the high specific surface area) or tend to aggregate, due to sintering of the de-hydroxylated particles (giving MK with the low specific surface area)^21^.
3. Concerning the sintering possesses, it is known that ZnO has a sintering retardation effect, i.e., ZnO hinders the grain growth of Al_2_O_3_^68^. Accordingly, the addition of ZnO particles is expected to hinder the growth of metakaolinite grains by limiting the rate of migration of MK grain boundaries to a value less than the rate of migration of pores, which then promotes the migration of pores^69,70^. Or better to say that ZnO particles, which were bonded to MK grains, form a strong network around the grains, hindering grain growth^71^. This action will produce ZnO doped MK with decreased average grain size and increases specific surface area compared to the undoped MK sample. Hence, ZnO controls the sintering behavior and microstructure of MK.

(ii) The mechanism of surface modification of MK by ZnO inside the cement pore solution (Fig. 11) may be similar to the coupling agent modification of kaolin by titanate^72^, as illustrated in the following steps:
  1. The dehydroxylated kaolinite still maintains residual hydroxyls (Eq. 4) even at higher temperatures of calcination:

Hence, the produced MK composes an alumina octahedral sheet and silica tetrahedral sheets^73^ having Si-O- and Al-O- surface groups that hydrolyze in the cement pore solution, forming Si-OH and Al-OH groups.

4 $$S{i_2}{O_5}A{l_2}{\left( {OH} \right)_4} \to S{i_2}{O_5}A{l_2}{O_{2 - x}}{\left( {OH} \right)_x} + \left( {2 - x/2} \right){H_2}O$$

 

(2) The surface of ZnO particles easily hydrolyzes in the pore solution of a cementitious matrix at pH > 12, forming a solid zinc hydroxide layer [Zn(OH)_2(S)_] which further dissolves (Eqs. 5 and 6), forming zincate ions^74^:
(3) The ZnO particles containing zincate ions Zn(OH)^-^ and Zn(OH)^2-^ form hydrogen bonding with Si-OH and Al-OH groups in the hydrolyzed MK surface.
(4) The dehydration of the hydrogen bonding between zincate ions and Si-OH and Al-OH groups results in the formation of Si-O-Zn(OH)_3_^2-^ and Al-O-Zn(OH)_3_^2-^ bonds.
(5) The electrostatic repulsive forces arising from the negatively charged surface stabilizes and prevents the MMK particles from aggregation^75^. Accordingly, coating the surface of MK particles with ZnO controls the surface chemistry of MK particles and enhances its dispersion in the cement matrix without agglomeration.

Taking into consideration that the addition of fine MK to concrete may result in a large agglomeration of MK particles that were not easily deflocculated during the batching of the concrete, and reduces the mechanical properties and performance of concrete^76,77^. Agglomeration mainly arises from the high specific surface area of MK and the Van der Waals electrostatic attractive force acting between cement and MK particles^7^.

5 $$Zn{\left( {OH} \right)_{2(S)}} + O{H^ - } \to Zn{\left( {OH} \right)_3}{^ - _{(aq)}}$$

6 $$Zn{\left( {OH} \right)_{2(S)}} + 2O{H^ - } \to Zn{\left( {OH} \right)_4}{^{2 - }_{(aq)}}$$

To the best of the knowledge of the author, the role of ZnO in improving the pozzolanic properties of MK was not adequately or convincingly explained in previous studies on this subject. Also, the mechanism derived from this study is novel and not addressed by any of the former researchers. The proposed mechanism was derived from observations of the microstructure of ZnO-modified MK and was supported by the individual properties of surface chemistry of MK and ZnO derived from their action in similar environments.

## Conclusions

The aim of this work is to clarify the interaction of ZnO with MK to explain the role of ZnO in improving the pozzolanic activity of MK. XRD and FTIR results of MMK did not detect products of the interaction of ZnO and kaolin due to the detection limits, because of the low added ZnO wt%. SEM results show the formation of non-aggregated MMK particles. The physico-chemical properties, strength activity index, FTIR, and XRD results indicated the higher pozzolanic activity of MMK. SEM results show the dispersion of cement particles coated with intense honeycomb-like C-S-H without being agglomerated inside the matrix of MMK blended cement mortars. ZnO improves the pozzolanic activity by modifying the surface properties of MK during the calcination process as well as during the hydration process.

ZnO particles modify the surface of MK during the calcination step by bonding to the aluminosilicate groups of the MK surface, producing MK particles coated with a ZnO layer. ZnO particles hinder grain growth and aggregation of MMK particles and produce MMK particles with decreased average grain size and increased specific surface area. ZnO particles modify the surface of MK during the hydration process by hydrolysis in the cement pore solution, forming zincate ions that hydrogen bond with Si-OH and Al-OH groups in the hydrolyzed MK surface, then form negatively charged species of Si-O-Zn(OH)_3_^2-^ and Al-O-Zn(OH)_3_^2-^ that repel and prevent the MMK particles from aggregation in the cement matrix.

MMK improves reactivity and dispersion, producing a denser C–S–H and lower portlandite content that is expected to have practical benefits beyond mechanical performance, such as reduction of porosity and permeability — likely enhancing durability against chlorides, sulfates, and CO₂. This makes it promising for durable blended cements, repair mortars, and potentially functional coatings (photocatalytic/antimicrobial). Targeted durability and functional tests (e.g., chloride migration, water absorption, photocatalytic/antimicrobial trials) are recommended for future studies to confirm field performance.

## Data Availability

The datasets used and/or analysed during the current study available from the corresponding author on reasonable request.• Data presented in the manuscript are instrumentation results (XRD, XRF, SEM).
